# Aicardi‐Goutières Syndrome Type 6: Case Report and Structural Prediction Supporting a Dominant‐Negative Effect of the *ADAR1* c.*3019G*>A Variant

**DOI:** 10.1002/ccr3.72146

**Published:** 2026-04-03

**Authors:** Katerina Turan, Petra Pokorna, Kamila Rihova, Jana Kubatova, Katerina Kozelkova, Eliska Hlouskova, Pavlina Danhofer, Petr Jabandziev, Jiri Damborsky, Regina Demlova, Ondrej Slaby, Katerina Slaba

**Affiliations:** ^1^ Department of Pediatrics, Faculty of Medicine, University Hospital Brno Masaryk University Brno Czech Republic; ^2^ Center of Precision Medicine Masaryk University Brno Czech Republic; ^3^ Department of Biology, Faculty of Medicine Masaryk University Brno Czech Republic; ^4^ Central European Institute of Technology (CEITEC) Masaryk University Brno Czech Republic; ^5^ CREATIC, Faculty of Medicine Masaryk University Brno Czech Republic; ^6^ Department of Pediatric Neurology, Faculty of Medicine, University Hospital Brno Masaryk University Brno Czech Republic; ^7^ Loschmidt Laboratories, RECETOX, Faculty of Science Masaryk University Brno Czech Republic; ^8^ International Clinical Research Centre St. Anne's University Hospital Brno Czech Republic

**Keywords:** ADAR1, Aicardi–Goutières syndrome type 6, dominant‐negative effect, structural prediction

## Abstract

Aicardi–Goutières syndrome type 6 (AGS6) is a genetically determined autoinflammatory disorder, classically inherited in an autosomal recessive manner. We report a Czech child with a heterozygous *ADAR1* NM_001111.5:c.3019G>A variant causing AGS6. In silico analysis supports a dominant‐negative effect, underscoring the need to consider dominant inheritance in AGS6 diagnosis and management.

**Abbreviations**
AD: autosomal dominant (inheritance)
AGS: Aicardi–Goutières syndrome
ANZCTR: Australia and New Zealand Clinical Trials Registry
AR: autosomal recessive (inheritance)
cDNA: complementary DNA
cGAS: cyclic GMP–AMP synthase
CSF: cerebrospinal fluid
CTIS: European Union's Clinical trials information system
ds: double‐strand (nucleic acid)
DSH: Dyschromatosis symmetrica hereditaria
HSP: hereditary spastic paraplegia
IFN: interferon
IFNAR: interferon alpha receptor
IFN‐I: type I interferons
IRF: interferon regulatory factor
ISGF: interferon‐stimulated gene factor
ISRE: interferon‐stimulated response elements
JAK: Janus kinase
LINE‐1: long interspersed nuclear elements
MAVS: mitochondrial antiviral signaling protein
MDA5: melanoma differentiation‐associated protein 5
MRI: magnetic resonance imaging
RIG‐I: retinoic acid‐inducible gene I
rWGS: rapid whole genome sequencing
SD: standard deviation
STAT: signal transducer and activator of transcription
STING: stimulator of interferon genes
TBK1: TANK‐binding kinase 1
TORCH: group of congenital infections
TYK2: tyrosine kinase 2
WHO‐ICTRP: World Health Organization International Clinical Trials Registry Platform

## 1 Introduction

Aicardi–Goutières syndrome (AGS) is a rare multisystemic inherited disorder primarily affecting the immune system and central nervous system. It was first described in 1984 by French pediatric neurologists in eight children from five families who developed progressive encephalopathy during the neonatal or early infancy period. The clinical presentation, mimicking congenital infection, included chronic lymphocytosis in cerebrospinal fluid (CSF), intracranial calcifications, and leukodystrophy, while serological tests for common congenital infections (TORCH) were negative [1]. According to the Orphanet database, over 500 cases of AGS with variable phenotypic manifestations have been published to date. Based on the underlying molecular genetic etiology, the condition is classified into nine types according to the causative gene [2]. However, due to limited awareness, broad phenotypic heterogeneity, and diagnostic challenges, the true prevalence of AGS is presumed to be higher than currently reported [3, 4].

A shared pathogenic mechanism across all AGS subtypes (AGS1–AGS9) is aberrant activation of interferon signaling, resulting in chronic autoinflammatory damage [2, 5]. Excessive type I interferon production, particularly IFN‐α, exerts neurotoxic effects [6], making central nervous system involvement of varying severity and character a hallmark of the clinical picture. Classical forms manifest as progressive neurological symptoms such as microcephaly, epilepsy, psychomotor delay or regression, dystonia, sleep disturbances, or neonatal seizures [7]. Diagnostic clues include findings from CSF analysis, serum markers, and neuroimaging. Characteristic features are chronic lymphocytosis in CSF, elevated IFN‐α and pterins in serum and CSF, and MRI findings of leukodystrophy, intracerebral calcifications (especially periventricular and in the basal ganglia), cerebral atrophy, or vasculopathy [8, 9, 10, 11]. A frequent clinical feature is the presence of painful, intermittently appearing, pruritic pernio‐like skin lesions, typically located on acral parts of the limbs [12], which may even represent the sole manifestation of the disease in some patients [13]. AGS may also present with nonspecific features of chronic disease such as hepatosplenomegaly, thrombocytopenia [14], endocrinopathies [15], feeding difficulties [16], or ocular abnormalities. Symptom onset most commonly occurs during the first year of life, but cases with adolescent or adult onset have also been reported [17].

Adult cases range from mild forms to severe manifestations, including isolated psychological symptoms or spastic paraplegia [15, 18, 19]. AGS is typically a disabling disorder with a prognosis depending on the specific genetic subtype and severity of clinical involvement. Even when the genetic cause is identified, predicting the disease course remains difficult. While some patients achieve a stable condition, neurological deficits often persist, increasing the patient's dependence on care [20].

Due to its heterogeneous clinical presentation, AGS diagnosis is often delayed and requires a high index of suspicion, particularly in individuals presenting with a phenotype resembling congenital infection in the absence of confirmed infectious agents. Clues such as progressive neurological regression, spastic paraparesis or quadriparesis, microcephaly, or characteristic skin lesions should prompt further investigation. Biochemical abnormalities in CSF, including elevated neopterin and IFN‐α levels, lymphocytic pleocytosis, and elevated protein content, can support clinical suspicion. Interpretation of neuroimaging findings depends heavily on the modalities used [21, 22, 23]. A definitive diagnosis, therefore, relies on molecular genetic testing.

In the AGS6, which is the focus of this case report, three cases have been described in the literature to date involving a heterozygous pathogenic variant in *ADAR1* identical to the one identified in our patient [24]. ADAR1 plays a critical role in maintaining immune tolerance to self RNA, and its dysfunction, including loss of its A‐to‐I RNA editing activity, results in inappropriate activation of antiviral pathways. The presumed pathogenic mechanism involves a dominant‐negative effect, in which the product of the mutated allele not only lacks normal function but also interferes with the function of the wild‐type protein. This mechanism may result in a clinical phenotype resembling that of an autosomal recessive disease. The hypothesis of a dominant‐negative mechanism for this specific pathogenic variant has been described in the literature [25]. However, this has not yet been confirmed experimentally nor supported by advanced molecular modeling. In this report, we present the case of a 13‐month‐old boy with progressive spasticity and psychomotor regression who was diagnosed with AGS type 6 due to a heterozygous *ADAR1* NM_001111.5:c.3019G>A variant.

## 2 Case History and Examination

**FIGURE 1:**
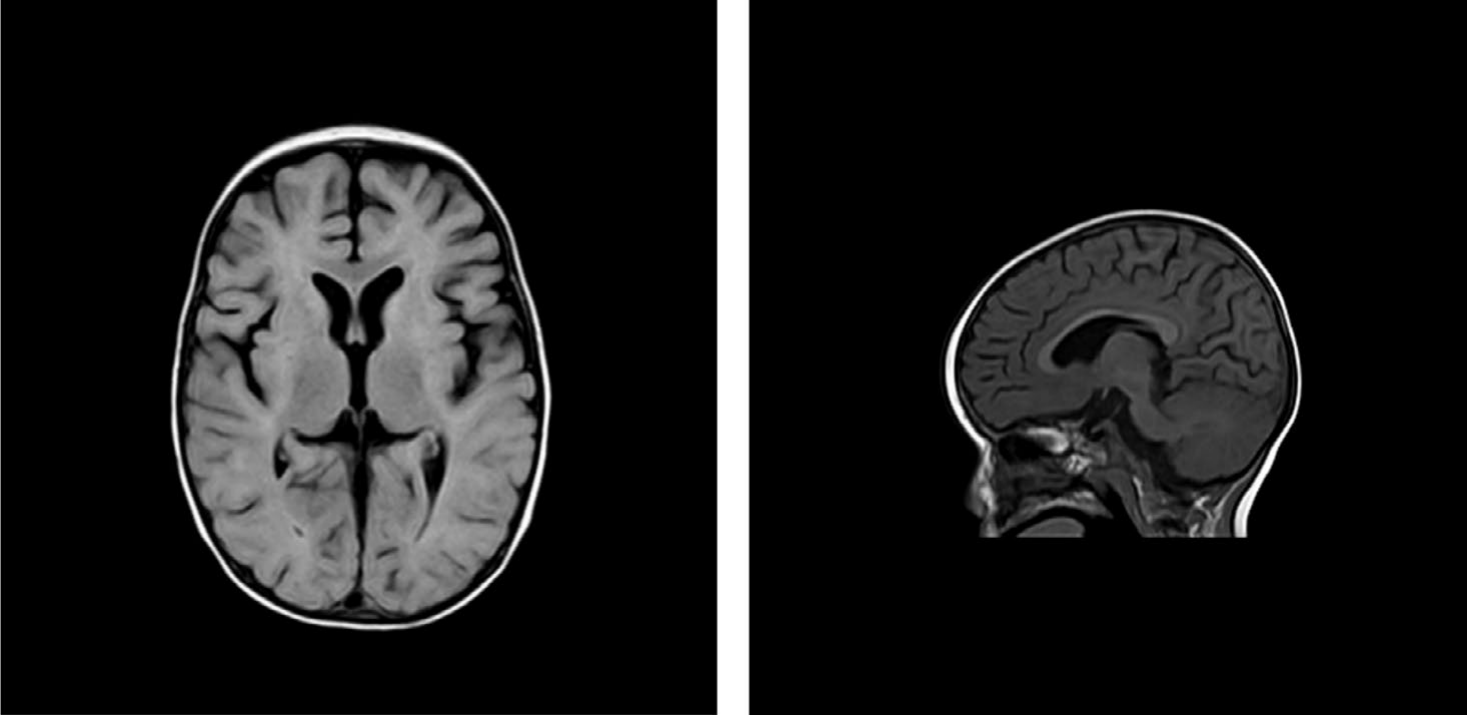
FIGURE 1 Brain MRI performed at 13 months of age. The examination showed deepened cerebral sulci suggesting cerebral atrophy, enlarged ventricular system, dysgenesis of the corpus callosum, and age‐appropriate myelination.

**FIGURE 2:**
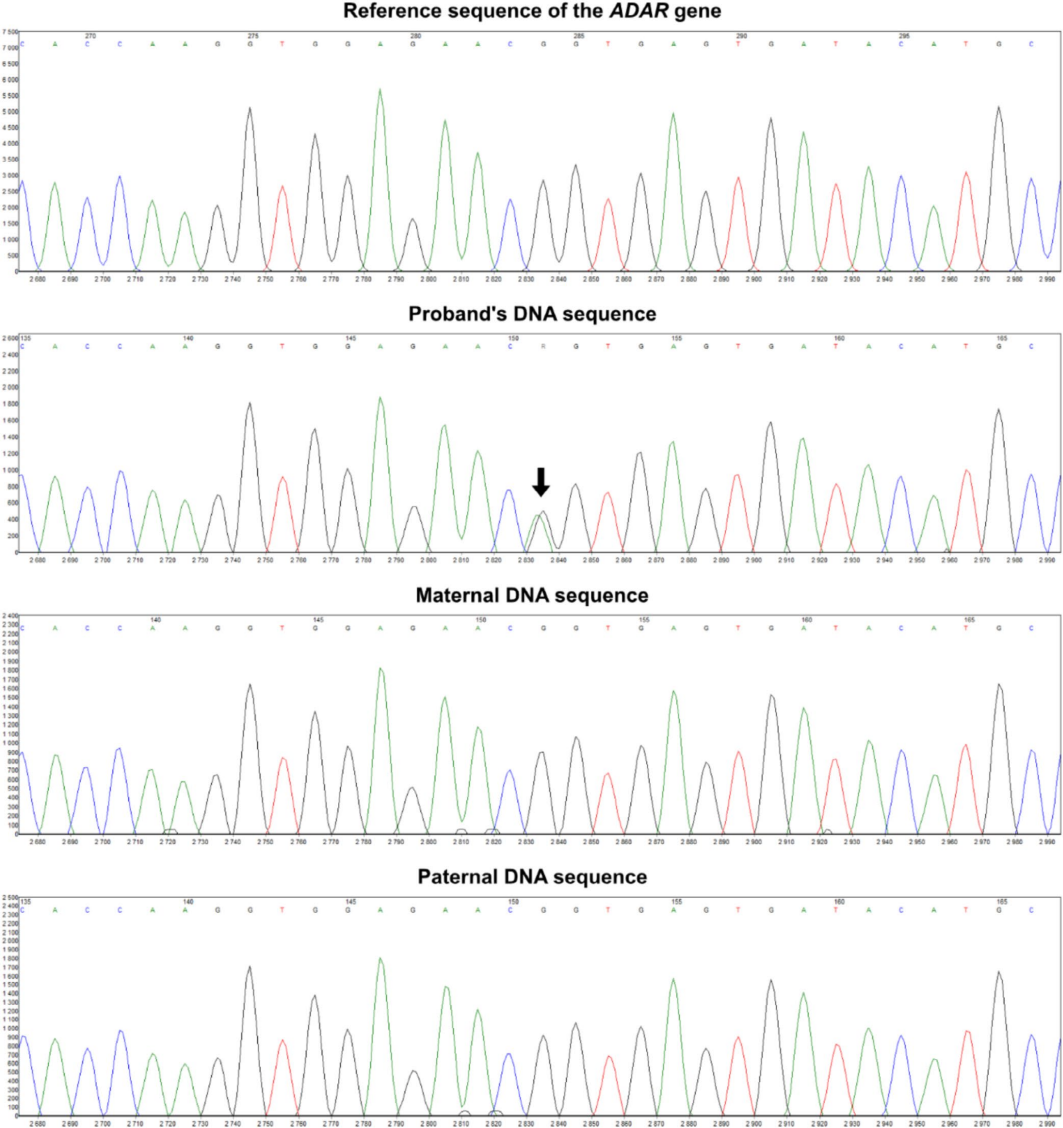
Electrophoretogram depicting the results of Sanger sequencing verification of the *ADAR1* NM_001111.5:c.3019G>A variant in the proband and both parents. A heterozygous G>A peak marked with an arrow is observed exclusively in the proband, with wild‐type sequence in both parents, confirming the variant's de novo origin.

We present the case of a 13‐month‐old boy referred for evaluation due to progressive spastic paraplegia evolving into quadrispasticity, accompanied by the regression of psychomotor development. Family history was negative, and the patient's sibling was healthy. The boy was born from the second pregnancy of a healthy mother. The third trimester of pregnancy was complicated by gestational hypertension treated with methyldopa. Prenatal ultrasound examinations revealed no abnormalities. Delivery was spontaneous at term, with the newborn being eutrophic and exhibiting an uneventful postnatal adaptation. Early psychomotor development was initially assessed as harmonious and within the broader range of normal. According to the parents, the child had a calm temperament since birth. However, around 6 months of age, he developed marked irritability, progressive spasticity, and regression of both motor and cognitive development. At the first neurological examination, quadrispasticity was observed, with developmental regression corresponding to the level of a neurologically impaired newborn. Somatic examination revealed microcephaly, general hypotrophy, hypogonadism, cryptorchidism, and micropenis. The parents reported difficulties with the introduction of solid foods, the patient was unable to chew and swallowed pieces whole. His diet remained limited to milk formula and blended food. At the age of 13 months, the boy was referred to our department for further evaluation of his overall health status. Anthropometric measurements showed a body length of 71 cm (2nd percentile, standard deviation [SD] –2.08), weight of 7.9 kg (0.6th percentile, SD –2.52) the weight‐for‐height ratio was within 19th percentile, SD –0.88. Head circumference reached 44 cm (2.1 percentile, SD –2.03). Brain MRI revealed dysgenesis of the corpus callosum with dilatated ventricular system and deepened cerebral sulci corresponding with mild cortical atrophy, and a laminar adenohypophysis with a thinned infundibulum and hypoplastic dimensions for age (< 2 mm) (Figure 1). Laboratory testing showed mildly elevated lactate (3.4 mmol/L), lactate dehydrogenase (5.73 μkat/L), and ALT (1.23 μkat/L). The MRI finding of a hypoplastic pituitary correlated with laboratory‐confirmed deficiency of gonadotropins and growth hormone. EEG and EMG did not reveal clear pathological findings. Given the polymorphic, rapidly progressive, and syndromic neurological presentation, the patient was enrolled in the national BabyFox study, a rapid whole‐genome sequencing (rWGS) program for seriously or critically ill children, performed in a trio setting (proband and both parents). Whole genome sequencing was performed using the Illumina NovaSeq 6000 system; variant calling was conducted through the DRAGEN pipeline V4.3, and tertiary analysis, including HPO‐driven phenotypic prioritization and clinical variant interpretation, was carried out using the Emedgene V36.2 platform (all solutions from Illumina Inc. San Diego, CA, USA). Pathogenic *de novo* heterozygous variant NM_001111.5:c.3019G>A, p.(Gly1007Arg) in the *ADAR1* gene was identified within 5 days of blood collection and subsequently verified by Sanger sequencing (Figure 2).

## 3 Differential Diagnosis, Investigations, and Treatment

Pathogenic variants in *ADAR1* are associated with autosomal recessive Aicardi–Goutières syndrome type 6 (MIM: 615010) and autosomal dominant *dyschromatosis symmetrica hereditaria* (DSH, MIM: 127400). Several recent studies have highlighted that probands with *de novo* heterozygous variants in *ADAR1* may present with features resembling spastic paraplegia, hypothesizing a dominant‐negative effect of the pathogenic variant [25, 26].

**FIGURE 3:**
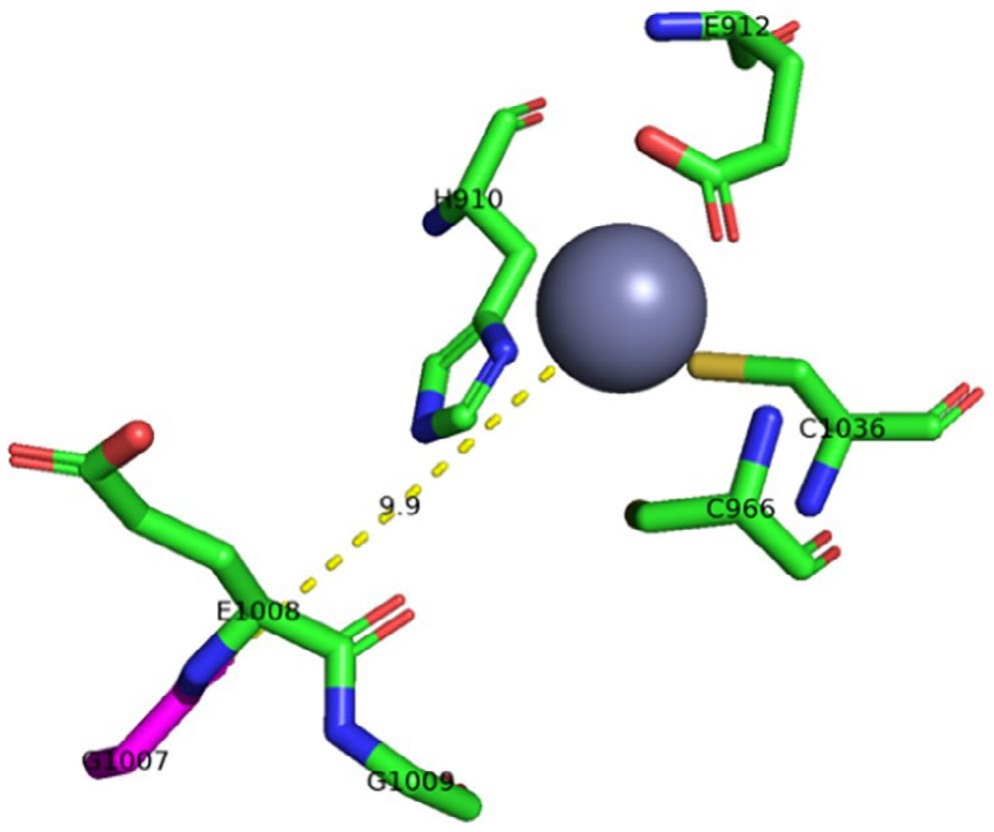
Position of the variant G1007R near the catalytic center of ADAR1. The key residues are shown in stick, while the coordinated catalytic zinc ion is shown as a sphere. The mutated residue G1007 (in purple) is located just next to the R1009 in the GRG loop, which is critical for RNA binding and catalysis. G1007 is at a 9.9 Å distance from the Zn^2+^ (yellow dashed line), which is coordinated by the residues H910, E912, C66 and C1036. The variants G1007R could increase the affinity of the mutant ADAR1 toward the negatively charged phosphate backbone of dsRNA due to an extra positive charge. The structure of ADAR1 was prepared using AlphaFold3 (DeepMind/Google), and the zinc ion was placed from the structure PDB ID 9D5K. The figure was prepared by PyMol.

To further support the hypothesis of a dominant‐negative effect of the pathogenic variant, we performed advanced molecular modeling (Figure 3). The G1007 residue in ADAR1 is located within the enzyme's catalytic deaminase domain. G1007 is a part of the highly conserved GEG triplet sequence (G1007‐E1008‐G1009) that resides within the base‐flipping loop of the deaminase domain. This loop plays a critical role in the enzyme's ability to flip the targeted adenosine out of the RNA helix and into the active site where deamination occurs [26]. The two glycine residues flanking the catalytic glutamate (E1008) are essential due to their small size and conformational flexibility. These properties allow the crucial catalytic glutamate residue E1008 to intercalate into the minor groove of the dsRNA substrate, a necessary step for facilitating the flipping of the targeted adenosine base [26].

The substitution of glycine, a small and nonpolar amino acid, with arginine, a large and positively charged amino acid, at position 1007 will disrupt this base‐flipping mechanism. The bulky side chain of arginine is likely to cause steric hindrance, interfering with the loop conformation and its ability to insert into the minor groove. Moreover, the introduction of a positive charge at this location could alter the electrostatic interactions within the active site and with the dsRNA substrate. Depending on the rotamer of the introduced positively charged arginine R1007, it can form a salt bridge with negatively charged E1008, which would be detrimental to the activity of ADAR1.

The hypothetical dominant‐negative effect of the ADAR1 G1007R variant can be attributed to two mechanisms: (a) Mechanism I, **competitive inhibition** through potentially enhanced RNA binding by mutant protein [27], and (b) Mechanism II, the potential **formation of non‐functional heterodimers** of the mutant protein with the wild‐type protein [28]. Both the enhanced RNA binding leading to competitive inhibition and the formation of non‐functional heterodimers can contribute to the overall dominant negative effect observed in individuals with the G1007R variants. The mutant protein's potential to bind RNA with higher affinity could directly block the wild‐type enzyme, while its ability to form dysfunctional dimers could further reduce the pool of functional ADAR1 complexes. Currently available experimental data do not allow us to distinguish between the two proposed mechanisms, which must therefore be regarded as hypothetical. In addition, in silico modelling is limited by incomplete structural information in public databases and by the high uncertainty in predicting the relative arrangement of individual domains. Resolving the structure of the ternary complex with bound RNA, including the region carrying the variant identified in this study, would require extensive additional structural analyses, which lie beyond the scope of the present work.

Based on current knowledge, clinical presentation, structural impact of the p.Gly1007Arg variant in ADAR1, and hypothetical mechanisms of dominant‐negative effect, the diagnosis of AGS6 was established in this patient.

## 4 Conclusion and Results

The patient was referred to a multidisciplinary neurologic and pediatric internal care. Symptomatic treatment focusing on spasticity was initiated. As part of pediatric internal medicine follow‐up, dysphagia rehabilitation and stimulation testing of the hypothalamic–pituitary axis were indicated, with a view toward hormone replacement therapy and possible surgical correction of cryptorchidism. The family was offered genetic counseling as part of future parental planning.

## 5 Discussion

We present the case of a child with a complex spectrum of neurological and somatic manifestations, characterized primarily by progressive spasticity and psychomotor regression. Given the syndromic and rapidly evolving clinical picture, genetic testing was performed. Rapid whole genome sequencing (rWGS) within the BabyFox study, confirmed by Sanger sequencing, identified a heterozygous pathogenic *de novo* variant in the *ADAR1* gene (NM_001111.5:c.3019G>A; p.Gly1007Arg), previously associated with both AGS6 and DSH [29]. This case demonstrates that first‐tier rWGS can deliver a timely etiologic diagnosis in critically ill infants with unclear syndromic presentations, curtail unnecessary testing, and guide clinical management.

Although our patient did not present with the full phenotypic picture of either AGS6 or DSH, recent reports [29] describe a clinical phenotype resembling hereditary spastic paraplegia (HSP) in individuals with heterozygous *ADAR1* variants. The brain MRI findings (cortical atrophy, ventricular dilatation, and corpus callosum dysgenesis) were consistent with previously described neuroimaging features in AGS patients. While the absence of intracranial calcifications could raise diagnostic uncertainty, it is known that their detection may be challenging using conventional MRI, particularly in young infants. Imaging techniques such as computed tomography or susceptibility‐weighted imaging sequences exhibit significantly higher sensitivity for detecting calcifications but were not performed in this case due to the patient's age and clinical stability at the time of evaluation. No cutaneous signs of DSH, such as hypopigmented or hyperpigmented macules on the face or dorsal aspects of the extremities, were present in our patient. However, the absence of DSH‐like cutaneous manifestations in genetically confirmed cases of AGS6 has been documented and may reflect variable expressivity of *ADAR1*, influenced by genetic background and environmental factors. This hypothesis is supported by the significantly higher incidence of clinically evident DSH in East Asian populations compared to other ethnic groups [29].

In this case, the clinical presentation was not suggestive enough to raise early suspicion of AGS. The diagnosis was made based on genetic testing, prior to the availability of cerebrospinal fluid or peripheral interferon‐related biomarkers. Although measurement of IFN‐α and pterin levels in CSF was initially planned, these tests were not performed due to their limited availability and the invasive nature of lumbar puncture in a neurologically fragile child. The variant NM_001111.5:c.3019G>A (p.Gly1007Arg) in *ADAR1* is associated with incomplete penetrance and variable expressivity, both of which remain poorly understood and may reflect the influence of genetic modifiers. Current evidence suggests a dominant‐negative mechanism of pathogenicity, in which the mutant protein not only loses its physiological function but also interferes with the function of the wild‐type allele. Structural modeling performed using AlphaFold and PyMOL supports this hypothesis, revealing the location of the mutated residue near the catalytic site of the deaminase domain.

Therapeutic options for AGS remain limited despite advances in molecular diagnostics. The standard of care continues to focus on symptomatic management, primarily targeting spasticity, epilepsy, feeding difficulties, and other systemic manifestations. In the case of the patient presented in this report, a 13‐month‐old boy with rapidly progressive spasticity, developmental regression, and a *de novo* heterozygous pathogenic variant NM_001111.5:c.3019G>A in the *ADAR1* gene, treatment was initiated within a multidisciplinary care setting, focusing on the management of neurological and endocrine complications. The patient received symptomatic therapy, including central muscle relaxants, rehabilitation, dysphagia intervention, and planned hormonal substitution due to confirmed pituitary insufficiency. Targeted immunomodulatory therapy was not initiated, primarily due to the patient's young age, the lack of robust evidence in the pediatric population, and an unclear risk/benefit ratio.

In recent years, increasing attention has been directed toward the use of Janus kinase (JAK) inhibitors, particularly baricitinib and ruxolitinib, which interfere with the JAK/STAT signaling pathway, a key effector downstream of type I interferon (IFN‐I) signaling and the central pathophysiological mechanism in AGS. Published case reports, including one involving the same *ADAR1* variant (p.Gly1007Arg), have described a positive response to a combination of corticosteroids and ruxolitinib, supporting the hypothesis that targeting the downstream IFN‐I‐mediated inflammatory cascade may be beneficial in selected AGS6 cases [25]. At present, however, no active clinical trials are enrolling patients with *ADAR1* variants, according to regional or global databases (CTIS, ANZCTR, WHO‐ICTRP). Compassionate use programs with baricitinib (e.g., NCT01724580, NCT03921554) have been completed. Long‐term corticosteroid therapy may help alleviate nonspecific systemic symptoms but does not result in significant neurological improvement. Various alternative methods for managing spasticity have been described in isolated case reports, including deep brain stimulation, intrathecal baclofen pumps [30], or botulinum toxin injections [19], although the effects were either limited or not well defined. Epilepsy associated with AGS generally responds well to standard antiepileptic drugs [31]. Therefore, the treatment of AGS6 remains primarily supportive and symptom‐focused, and decisions regarding potential immunomodulatory interventions must be made on an individual basis considering patient age, disease severity, systemic inflammatory activity, and the availability of specialized centers.

To the best of our knowledge, this is the first genetically confirmed case of AGS6 with the c.3019G>A variant reported in a Czech patient. In the presented case, the diagnosis of AGS6 was established based on rapid molecular genetic testing, which identified a heterozygous pathogenic *ADAR1* variant (NM_001111.5:c.3019G>A; p.Gly1007Arg). Although most AGS subtypes follow autosomal recessive inheritance, AGS6 has been repeatedly associated with autosomal dominant transmission, likely mediated by a dominant‐negative effect of the mutant protein on the wild‐type allele. Our structural modeling supports this hypothesis, illustrating how the G1007R substitution may interfere with RNA binding and catalysis, and potentially disrupt the function of the wild‐type protein in heterozygous individuals. At present, care is focused on symptomatic management, while targeted immunomodulation, including JAK/STAT inhibition, remains investigational [32]. From a diagnostic perspective, AGS should be considered in the differential diagnosis of early‐onset neurological regression, particularly in cases mimicking congenital infection without identified pathogens or perinatal risk factors. Early genetic diagnosis can significantly influence clinical decision‐making, prognosis, and reproductive counseling.

## Author Contributions

**Katerina Turan:** data curation, writing – original draft. **Petra Pokorna:** formal analysis, investigation, methodology. **Kamila Rihova:** formal analysis, writing – review and editing. **Jana Kubatova:** data curation, writing – review and editing. **Katerina Kozelkova:** data curation, investigation. **Eliska Hlouskova:** formal analysis, investigation. **Pavlina Danhofer:** data curation, writing – review and editing. **Petr Jabandziev:** data curation, resources. **Regina Demlova:** data curation, writing – review and editing. **Jiri Damborsky:** investigation, software, visualization, writing – original draft. **Ondrej Slaby:** conceptualization, resources, supervision, writing – review and editing. **Katerina Slaba:** data curation, methodology, supervision, writing – original draft, writing – review and editing.

## Funding

Supported by the Ministry of Health of the Czech Republic – RVO (University Hospital Brno 65269705) and by the grant of the Faculty of Medicine, Masaryk University: MUNI/A/1591/2023. This publication is also the outcome of the project CREATIC funded from the European Union's Horizon Europe Coordination and Support Action under the Grant agreement number 101059788. Computational resources were provided by the CZECRIN and e‐INFRA projects (LM2023049 and 90254), supported by the Ministry of Education, Youth and Sports of the Czech Republic. The Baby Fox study, a national initiative focused on rapid whole‐genome sequencing (rWGS) in critically ill children, is conducted in collaboration with Illumina Inc., which provides reagents, bioinformatics solutions and support, through a Collaboration Agreement.

## Ethics Statement

The study was approved by the University Hospital Brno Ethics Committee (approval no. 12–071222), and informedconsent was obtained and signed by the patient's parents.

## Consent

Written informed consent was obtained from the patient to publish this report in accordance with the journal patient consent policy.

## Conflicts of Interest

The authors declare no conflicts of interest.

## Data Availability

Sequencing data are available from the corresponding author upon reasonable request.
